# An Integrated Vegetated Treatment System for Mitigating Imidacloprid and Permethrin in Agricultural Irrigation Runoff

**DOI:** 10.3390/toxics9010007

**Published:** 2021-01-09

**Authors:** Bryn M. Phillips, Michael Cahn, Jennifer P. Voorhees, Laura McCalla, Katie Siegler, David L. Chambers, Thomas R. Lockhart, Xin Deng, Ron S. Tjeerdema

**Affiliations:** 1 Department of Environmental Toxicology, University of California, Davis, CA 93940, USA; jpvoorhees@ucdavis.edu (J.P.V.); lmccalla@ucdavis.edu (L.M.); csiegler@ucdavis.edu (K.S.); rstjeerdema@ucdavis.edu (R.S.T.); 2 University of California Cooperative Extension, Monterey County, CA 93901, USA; mdcahn@ucanr.edu (M.C.); dlchambers@ucanr.edu (D.L.C.); trlockhart@ucanr.edu (T.R.L.); 3 California Department of Pesticide Regulation, Sacramento, CA 95814, USA; Xin.Deng@cdpr.ca.gov

**Keywords:** vegetated treatment system, biochar, granular activated carbon, polyacrylamide, imidacloprid, permethrin, pyrethroid, neonicotinoid, aquatic toxicity

## Abstract

Pyrethroid and neonicotinoid pesticides control an array of insect pests in leafy greens, but there are concerns about the off-site movement and potential water quality impacts of these chemicals. Effective on-farm management practices can eliminate aquatic toxicity and pesticides in runoff. This project evaluated an integrated vegetated treatment system (VTS), including the use of polyacrylamide (PAM), for minimizing the toxicity of imidacloprid and permethrin pesticides in runoff. The VTS incorporated a sediment trap to remove coarse particles, a grass-lined ditch with compost swales to remove suspended sediment and insecticides, and granulated activated carbon (GAC) or biochar to remove residual insecticides. Runoff was sampled throughout the VTS and analyzed for pesticide concentrations, and aquatic toxicity using the midge *Chironomus* *dilutus* and the amphipod *Hyalella azteca*. In simulated runoff experiments, the VTS reduced suspended sediment load by 88%, and imidacloprid and permethrin load by 97% and 99%, respectively. In runoff events from a conventionally grown lettuce field, suspended sediment load was reduced by 98%, and insecticide load by 99%. Toxicity was significantly reduced in approximately half of the simulated runoff events, and most of the lettuce runoff events. Integrated vegetated treatment systems that include components for treating soluble and hydrophobic pesticides are vital tools for reducing pesticide load and occurrence of pesticide-related toxicity.

## 1. Introduction

Growers rely on applications of pyrethroid and neonicotinoid pesticides for the control of an array of insect pests in leafy greens. Concerns about the off-site movement of agricultural chemicals in irrigation runoff and impacts to water quality have led to stricter governmental regulations and the eventual loss of registration (e.g., chlorpyrifos in California). Effective on-farm management practices are needed to reduce pesticide load and eliminate aquatic toxicity of pyrethroid and neonicotinoid pesticides in runoff. However, neonicotinoid pesticides are very water soluble and pyrethroid pesticides are very hydrophobic and are transported on suspended sediments in runoff. Management practices that can mitigate both water-soluble and insoluble pesticides are needed to minimize off-site impacts to water quality.

Both retention basins and vegetative treatment systems (VTSs) reduce suspended sediments and hydrophobic pesticides in runoff [1]. Retention basins can be permanent, engineered structures or temporary basins located downslope from a farmed area that capture runoff during the growing season and settle out large silt and sand-size particles suspended in runoff. Retention basins integrated in sequence with vegetated ditches within a VTS can reduce pyrethroid pesticides up to 100% [2]. Vegetated treatment systems are usually drainage ditches that have been planted with grass or similar vegetation that can slow the flow and infiltrate runoff from agricultural fields. Research has demonstrated that VTSs, used individually or as part of a suite of management practices, reduce pesticide loads in tailwater runoff [3,4,5,6,7]. Treating irrigation water with polyacrylamide (PAM), a long-chain polymer used for erosion control, can also reduce the suspended sediments in runoff by more than 90% [8,9]. The addition of compost and granulated activated carbon (GAC) to a grass-lined ditch has been shown to reduce the load of chlorpyrifos, an intermediately soluble pesticide, by up to 98% [7]. Integrated vegetated treatment systems have also been shown to reduce pesticide-associated toxicity to invertebrates in runoff [2,7]. While these systems are effective at reducing organophosphate and pyrethroid pesticides, they have not been evaluated for treating more soluble insecticides, such as the neonicotinoid imidacloprid. Imidacloprid is used in conjunction with pyrethroids on most lettuce crops in the Salinas Valley, and has recently been detected in waterways receiving runoff in the central coast region of California [10].

Because neonicotinoids are water soluble, they can be transported from application sites via surface water runoff and groundwater [11]. Neonicotinoids are systemic pesticides that are designed to be taken up by the plant [12,13], with a portion remaining in the soil, where it breaks down [14]. The fate of imidacloprid through plant uptake and soil degradation is well known, but because of its solubility, a significant amount of this chemical can be transported from the application site in runoff. The solubility of imidacloprid makes it resistant to treatment; therefore, two forms of activated carbon were tested to determine their effectiveness at reducing this neonicotinoid. Activated carbon filtration is commonly used in industrial applications as a method to remove organic compounds from wastewater and has been suggested for surface water treatment [15,16]. Biochar has been used as remediation for contaminated soils [17,18], and some recent research has been conducted applying biochar to treat pesticides in simulated agricultural runoff [19,20]. Recent research demonstrated the removal of chlorpyrifos by activated carbon in simulated agricultural runoff [7], and a laboratory study showed similar success with imidacloprid and chlorpyrifos removal [21].

The goal of this project was to evaluate the efficacy of an integrated VTS to mitigate chemical loading and related toxicity of a water soluble neonicotinoid (imidacloprid) insecticide and a hydrophobic pyrethroid (permethrin) insecticide. The VTS used in this study was built upon a design presented in Phillips et al. [7], and incorporated a sediment trap area to remove coarse particulates, a grass-lined ditch with compost swales to remove suspended sediment and insecticides, and a final treatment using GAC or biochar to remove residual pesticides. Polyacrylamide (PAM)-treated irrigation water was used in conjunction with these practices to minimize the suspended sediment concentration of the runoff. The VTS was evaluated with simulated irrigation flows spiked with imidacloprid and permethrin, as well as runoff from a conventionally grown lettuce field treated with these insecticides.

## 2. Materials and Methods

**Figure 1:**
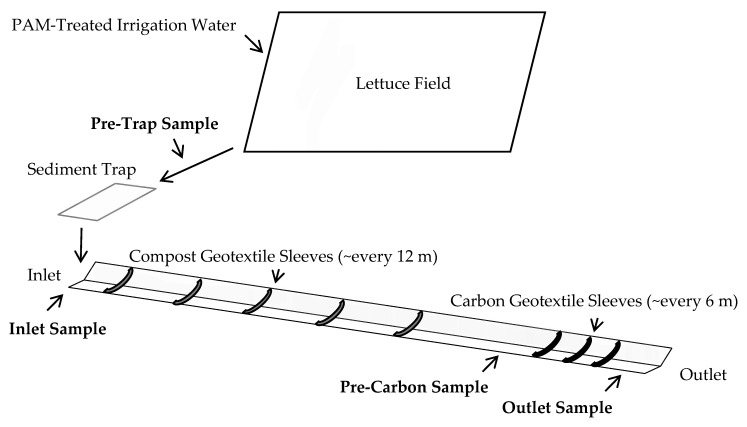
Schematic of ditch system (not to scale). Entire ditch was vegetated. Compost and granulated activated carbon (GAC) installations were as shown.

Field trials were conducted at the US Department of Agriculture-Agricultural Research Service (USDA-ARS) Spence Research Farm, located in Salinas, CA, USA. Trials evaluated the efficacy of an integrated VTS to reduce the concentration of permethrin, imidacloprid, suspended sediments, and aquatic toxicity in two phases as follows. Phase 1 field trials evaluated the combined efficacy of the vegetated ditch, compost swales, and GAC or biochar treatment using simulated runoff from overhead sprinklers. Phase 2 trials evaluated the combined efficacy of PAM, a sediment trap, vegetated ditch, compost swales, and biochar to mitigate these pesticides in runoff from a lettuce field irrigated with overhead sprinklers (Figure 1). The vegetated ditch used for these studies was 160 m long, 3 m wide, 1 m deep, with a 2–3% slope. The ditch was vegetated with mature red fescue grass (*Festuca rubra*) originally seeded in 2007. Six compost sleeves were placed at the upper end of the ditch, spaced approximately at 11 m intervals. Compost installations were constructed with 1.5 m lengths of permeable geotextile sleeve (Filtrexx^®^ SafteySoxx^®^, Grafton, OH, USA) filled with compost derived from green yard waste from a local supplier. The compost sleeves were oriented at the bottom of the vegetated ditch perpendicular to the direction of the flow. Two types of carbon material, namely granular activated carbon (GAC, Evoqua, Pittsburg, PA, USA) and biochar (AgraMarketing, Tracy, CA, USA), were tested as carbon filters during the trials. Three carbon filter installations were located at the lower end of the vegetated ditch at approximately 6 m intervals (Figure 1). The carbon filters were constructed using 1 m sections of Filtrexx SafteySoxx sleeves filled with either GAC or biochar. The carbon-filled sleeves were placed along the upstream side of a wooden board inserted perpendicular to the ditch.

In the first phase of the study, known concentrations of imidacloprid (4 mg/L) and permethrin (24 mg/L) were added to simulated runoff. Suspended sediment was added to the runoff by irrigating bare ground with 15 overhead sprinklers (20JH, Rainbird Inc., Tucson, AZ, USA) spaced on a 9 m grid, located approximately 160 m upslope from the inlet of the vegetated ditch. A portion of the sprinkler runoff was combined with the well water dosed with imidacloprid and permethrin using a gas-powered pump and a 7.6 cm diameter manifold made of PVC pipe. Valves on the manifold were adjusted to proportion the flow rate of the well water and sprinkler runoff to attain a final flow rate of approximately 285 L per minute and a turbidity between 200 to 300 NTU.

Concentrated solutions of imidacloprid and permethrin were prepared fresh for each trial by adding a certified reference material to a known volume of distilled water (Accustandard, New Haven, CT, USA). Stock solution concentrations were calculated to yield inlet concentrations of approximately 4200 ng/L imidacloprid and 700 ng/L permethrin. Stock solution was injected into the manifold at a flow rate of 50 mL/min using a metering pump (Model MD, Seepex GmbH, Bottrop, Germany).

In the second phase of the study, runoff from a 0.8 ha lettuce field was treated. Romaine lettuce (cv. True Heart) was planted in two rows spaced 30 cm apart on 1 m wide beds on 17 September 2019. The seed was drenched with Admire^®^ Pro (imidacloprid) and Perm^®^-Up (permethrin) at rates of 767 and 584 L/ha, respectively, during planting. Anticrustant and Kerb^®^ (pronamide) herbicide were sprayed in bands over the seed lines on 18 September 2019. Additionally, the crop received a foliar application of imidacloprid and permethrin at the same rates used at planting on 6 November. The crop was germinated using overhead sprinklers starting on 19 September 2019. A flowmeter (Ag3000, Seametrics, Kent, WA, USA) was used to monitor irrigation water volume. Irrigation water applied to the trial area was treated with polyacrylamide (PAM) (Hydrosorb 100D, Aqua Ben Corporation, Orange, CA, USA) by diverting approximately 1/3 of the flow through a “dry PAM applicator”, described by Cahn [22] that conditioned the irrigation water with a low concentration of PAM (<1 ppm). Runoff from the lettuce field flowed through a sediment trap prior to entering the vegetated ditch portion of the VTS that measured approximately 15 m × 2 m with an average depth of 0.3 m. All carbon installations in the second phase of the study were composed of biochar.

Application of simulated runoff water to the vegetated ditch lasted between 3 and 3.5 h. Runoff events from the irrigated lettuce varied between 4.5 and 5 h. The flow rate of runoff entering the ditch in Phase 1 was monitored with a magnetic flowmeter (Ag2000, Seametrics, Kent, WA, USA) for determination of the total runoff. The flowmeter was wired to a datalogger (CR300, Campbell Scientific, Logan, UT, USA) to record the flow rate at 5 min intervals. The outflowing runoff was measured with a magnetic flowmeter (WMP101, Seametrics, Kent, WA, USA). The volumes of water entering and exiting the VTS during the Phase 2 trials were monitored with V-notch weirs equipped with stilling wells and a float mechanism calibrated to estimate the water height. A datalogger recorded the height of the float in the stilling well at 5 min intervals. The height values were transformed with a calibration curve to estimate the flow rate of the runoff exiting the weirs.

A datalogger was also used to activate peristaltic pumps (Omegaflex FPU-122-12VDC, Omega Engineering, Stamford, CT, USA) to collect composite samples of runoff during simulated and actual irrigation events of the Phase 1 and 2 trials. For the Phase 1 trials, pumps were located at the inlet of the ditch, upstream of the carbon installations (approximately 130 m from the inlet), and at the outlet (approximately 153 m from the inlet, Figure 1). For the Phase 2 trials, an additional sampling station was located upstream of the sediment trap. Pumps were activated for two minutes at 5 min intervals and collected approximately 400 mL per interval. Runoff was collected through a stainless-steel tube suspended in the center of the ditch and drawn through silicone tubing into a 12 L stainless-steel container. Composite samples from each location were transferred into amber glass bottles at the end of a trial and transported to the Marine Pollution Studies Laboratory (Monterey, CA, USA) for toxicity testing and turbidity measurements. Subsamples were shipped to the California Department of Food and Agriculture (CDFA) Laboratory (city) for pesticide analysis and measurements of suspended solids. Imidacloprid concentrations were measured using LC/MS (CDFA method EMOM-SM-05-037) with a method detection limit of 4 ng/L and a reporting limit of 10 ng/L. Permethrin concentrations were measured using GC/MS (CDFA method EMOM-SM-05-022) with a method detection limit of 0.8 ng/L and a reporting limit of 1 ng/L.

Toxicity was assessed using the amphipod *Hyalella azteca* and the midge *Chironomus dilutus*. Testing procedures followed modifications of U.S. EPA methodology [23]. These organisms were chosen based on their sensitivities to the pesticides being evaluated. The amphipod is more sensitive to pyrethroids, such as permethrin, whereas the midge is more sensitive to the neonicotinoids, such as imidacloprid. Permethrin median lethal concentrations (LC50s) for the amphipod and midge are 21.1 ng/L [24] and 99 ng/L [25], respectively, whereas the imidacloprid LC50s are 383 µg/L and 11.8 µg/L [26], respectively. Significant toxicity was determined statistically using a separate-variance *t*-test followed by a comparison to a threshold of 80% of the control response. Toxicity responses were compared to spiked chemical concentrations using the toxic unit (TU) approach. Toxic units (TUs) were calculated by dividing the measured concentration by the organism-specific LC50. One TU would be expected to cause a 50% effect on the organism.

Six runoff simulations were conducted in the vegetated treatment system between 25 March 2019 and 20 May 2019 (Table 1) for the Phase 1 trials. Trials compared the efficacy of biochar and granular activated carbon to remove pesticides from runoff by conducting six runoff events that were randomly assigned with either a GAC or biochar carbon treatment. Three runoff events from the sprinkler irrigated lettuce field were monitored for the Phase 2 trials between 24 September 2019 and 12 November 2019. The first two events followed the drenching application of Admire-Pro and Perm-up at planting, and the third event occurred six days after the foliar spray. Reductions in concentration were calculated by dividing the outlet concentration by the inlet concentration. Calculations of load reduction combined concentration reduction with infiltration. For the purposes of both calculations, non-detects were assumed to be equivalent to zero.

## 3. Results

Total runoff entering the ditch averaged 55,997 L during the Phase 1 simulation trials and outflow averaged 16,922 L. Infiltration rates were calculated from the difference between inflow and outflow and averaged 70% of the inflow volume (Table 1), with variability likely related to the saturation of the ditch before each trial. Measured inlet concentrations of imidacloprid ranged from 76% to 114% of the target dosing concentration, whereas measured permethrin concentrations ranged from 23% to 42% of the target dosing concentration. Measured concentrations of imidacloprid were close to target concentrations because the insecticide is relatively soluble, whereas permethrin is hydrophobic and tends to associate with particles and surfaces. Spiking concentrations were chosen knowing that there would be substantial loss of permethrin in the stock solution pump and irrigation manifold.

Imidacloprid concentrations were reduced by 45–76% in the VTS prior to the carbon filtration, which further reduced the concentrations by a total of 88–94% (Table 1). Imidacloprid is highly soluble (log K_ow_ = 0.57) and is less likely to bind to the grass or the compost installations; therefore, it was necessary to have a carbon component to achieve greater than 90% reduction. Permethrin concentrations were reduced by 87–99% in the VTS prior to carbon filtration, which increased reduction up to 91–100%. Permethrin is hydrophobic and less soluble (log K_ow_ = 6.5), and is more likely to associate and bind to plant material and compost. There was no difference in the treatment effectiveness between GAC and biochar. Load reduction in this system ranged from 94% to 100%. Concentration and load reductions in this system were similar to those previously reported for chlorpyrifos [7].

Suspended sediment concentrations entering the VTS averaged 180 mg/L (Table 1). The relatively low concentrations of suspended sediment at the inlet were intended to simulate a pretreatment of the irrigation water with a sedimentation basin or PAM, which has been shown to reduce suspended sediment concentration in sprinkler runoff by 90% [8]. The load reduction in sediment was calculated from the flow data and sediment concentration at the inlet and outlet of the VTS. The load reduction of suspended sediment averaged 90%.

Inlet samples were significantly toxic to both *H. azteca* and *C. dilutus* (Table 2). The VTS was able to remove *H. azteca* toxicity from one sample, and remove *C. dilutus* toxicity from five samples. Midge survival generally tracked with imidacloprid and permethrin TUs, but amphipod survival remained low in the majority of samples. Permethrin concentrations were generally reduced well below the toxicity threshold for the amphipod (~0.5 TU), and imidacloprid concentrations never exceeded a tenth of a TU. Because neither of the spiked chemicals were measured at concentrations toxic to the amphipod, the CDFA lab investigated the presence of other pyrethroids in the simulated runoff samples. Because the experiment involved simulated runoff from other areas of the research farm, it was possible this runoff contained pesticides from previously cultivated crops. This could have been particularly true of pyrethroids being mobilized with the suspended sediments added to the simulated runoff. It was confirmed that bifenthrin was present in all samples tested for the 14 May trial, but the concentrations were not quantified. Bifenthrin concentrations were quantified in the 20 May samples, which contained approximately 0.9, 0.7, and 0.3 TUs at the inlet, pre-carbon station, and outlet, respectively.

Midge survival was significantly improved by the pre-carbon section of the VTS in five of the six simulated irrigation trials, and growth was improved in all trials where measurements were available (Table 2). Toxic unit values in the inlet water for both insecticides ranged from 0.56 to 2.99, and were reduced to less than 0.4 in all trials. Although spiked insecticide concentrations were below toxicity thresholds, significant toxicity was observed in the pre-carbon and outlet samples during the 20 May trial. The cause of this toxicity is not known, but it is possible that another pesticide, not intended to be in the runoff, was present in the ditch or in the sediment trap at the time of testing. Perhaps the same unmeasured pyrethroids that were contributing to amphipod toxicity could have had high enough concentrations to cause midge toxicity. In addition, significant volumes of storm runoff from adjacent strawberry fields flowed through the sediment trap and through the ditch less than 15 days before the trial.

The second phase of the study monitored the treatment of actual runoff from a 0.8 hectare lettuce field. In addition to the three samples collected in the VTS (inlet, pre-carbon, and outlet) a fourth sample was collected before the sediment trap (pre-trap). Irrigation water was also pre-treated with PAM to decrease the suspended solid concentrations entering the VTS. An error occurred when retrieving flow data from the dataloggers resulting in the loss of water volume data for the first two monitored irrigation events. However, because similar flow characteristics were observed among all runoff events, data from the third monitored event were used to estimate load reductions in the first two events. Irrigation application volume was also recorded as part of the third irrigation event.

**Figure 2:**
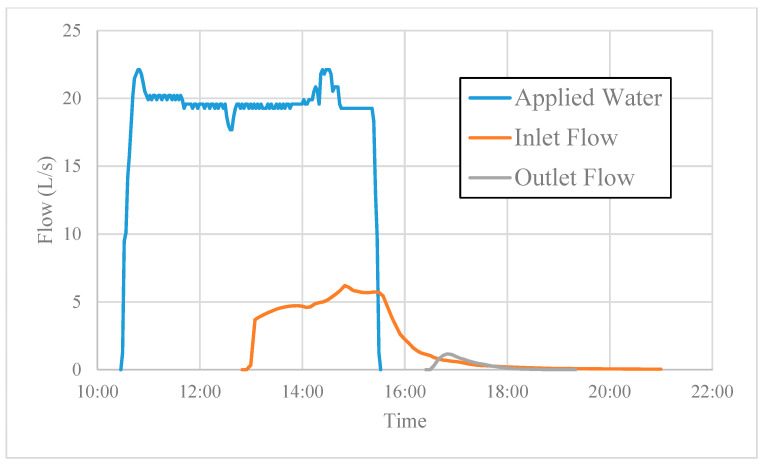
Flow rate of applied water from sprinklers and of runoff entering and exiting the vegetated treatment ditch.

Reductions of insecticide and suspended sediment concentrations were calculated based on comparing pre-sediment trap concentrations to those at the outlet of the VTS. Water flow was measured at the inlet to the vegetated portion of the system, and not at the pre-sediment trap location. It was assumed that a negligible volume of water infiltrated in the sediment trap; therefore, load reductions were calculated using input water volume measurements and concentrations detected in the pre-trap sample. During the third irrigation event, 348,301 L of water were applied to the lettuce field (Figure 2). Approximately 17% of this volume was measured as runoff into the VTS (58,211 L). Of the water that entered the VTS, 94% infiltrated.

Inclusion of PAM in the irrigation water, and the addition of a sediment trap upstream of the vegetated portion of the system reduced the particle load of the runoff by an estimated average of 45% before water flowed into the vegetated portion of the system. Because permethrin is hydrophobic and associates with sediments, the pre-vegetation reduction of sediment reduced permethrin concentrations by an average of 49%. Reduction of sediment load did not reduce concentrations of imidacloprid due to its solubility. Suspended solids were further reduced in the vegetated portion of the VTS by an average of 81%, with final load reductions averaging 98%, which translated to average sediment reductions of 19 kg per irrigation event.

The effectiveness of PAM treatment of the irrigation water was not specifically measured as part of this study, but presumably minimized the suspended sediment concentration of the runoff leaving the field. Past trials have shown that suspended sediment concentrations in sprinkler runoff at the Spence Research Farm are typically greater than 1000 mg/L. The sediment concentration of the runoff for these three sprinkler events averaged 318 mg/L entering the sediment trap and 181 mg/L at the inlet (Table 3). Concentrations of suspended sediment continued to decrease as the runoff flowed through the vegetated ditch averaging 60 mg/L at the outlet of the ditch.

Target spiking concentrations in the first phase of the study were high enough to not only cause toxicity to the test organisms, but also high enough to demonstrate reduction through treatment. Concentrations were also chosen based on those measured in natural systems receiving runoff from agricultural areas. Concentrations of imidacloprid and permethrin measured during the first two trials of the second phase of the study were approximately five times lower than the average concentrations spiked during the first phase (Table 3). The third irrigation event of the second phase, which followed the foliar application of the insecticides, produced concentrations of imidacloprid in the runoff water that were approximately six times higher than concentrations spiked during the first phase. The VTS as a whole reduced imidacloprid and permethrin concentrations in runoff by an average of 76% and 93%, respectively, (Table 3) and loads of both insecticides were reduced by 99%. Based on water concentrations and calculated load reductions, it is estimated that as much as 465 g of imidacloprid and 3 g of permethrin were removed from runoff during the three irrigation events.

As during the first phase of the study, runoff from the lettuce field contained high enough concentrations of insecticides to cause significant mortality to both test organisms, and significant growth effects to *C. dilutus* (Table 4). Although samples collected at the outlet of the system were often still significantly toxic, survival markedly improved in all of the *H. azteca* samples and two of the three *C. dilutus* samples. Permethrin concentrations presented as TUs tracked fairly well with *H. azteca* survival. Midge survival in the 24 September and 8 October trials did not track with concentrations of either insecticide, and was likely influenced by unmeasured chemicals, but both trials demonstrated significant treatment. Application of PAM was discounted as a cause of toxicity because this formulation is not toxic to the organisms tested [27]. The 11 November trial had a high magnitude of toxicity due to an elevated concentration of imidacloprid in the runoff, and although the concentration of imidacloprid was reduced by 74%, no reduction of toxicity was possible.

## 4. Discussion

Vegetated treatment systems are generally able to reduce chemical loads to some degree [2,3,4,6,28,29,30], but the addition of key treatment components to the current system enabled further load reduction for both hydrophobic and soluble insecticides. The previous study on this system demonstrated an average of 96% load reduction of chlorpyrifos with vegetation and carbon [7], but the addition of a PAM application and sediment trap further reduced particle and insecticide loads by 97–100%. Because of the difficulty in treating soluble pesticides [2,30], use of a carbon installation is necessary to reduce loading of insecticides such as imidacloprid.

The efficacy of the PAM addition was not determined in this study, but a previous study by Cahn et al. [8] demonstrated that the addition of this polymer can reduce suspended sediment concentrations in runoff by as much as 90%. A concurrent field study evaluating the treatment of irrigation water with PAM, similar to the approach evaluated in the Phase 2 trials, demonstrated an average reduction of sediment concentration in runoff of 87% on the same soil type as this study [22]. In the current study, the combined use of PAM and a sediment trap reduced the particle loads by almost half before the water entered the vegetated portion of the VTS. Larger sediment basins with longer retention times can also reduce a significant amount of the particle load [31], but take up more space at the edge of field. Reducing suspended sediment concentrations prior to having water enter the integrated VTS with compost and carbon installations will prolong the operational life of the system by minimizing clogging and deposition of large-sized particles.

Phillips et al. [7] previously studied GAC in this integrated VTS, but the current study compared the effectiveness of GAC and biochar and found no differences. Biochar is a collective term for carbon products produced by heating biomass in a closed system with little or no air [32]. These substances are traditionally used as soil amendments, and have been used successfully as remediation or treatment of contaminated soils [17,18], drinking water [33,34], and pesticides in simulated agricultural runoff [19,20]. Biochar is less expensive than GAC (<$1 per pound vs. $2 per pound for GAC), and can be disposed onsite by applying to an agricultural field rather than having to be hauled away. No studies have demonstrated the long-term efficacy of biochar in a field setting, but Voorhees et al. [21] attempted to overwhelm biochar in laboratory experiments with chlorpyrifos and imidacloprid. A scaled-up version of their experiment, which was used for the carbon installation in the current study, would treat above-average runoff from approximately 24 hectares of central California-grown lettuce for one season.

In the first study using this integrated VTS, Phillips et al. [7] were not able to demonstrate that the system could reduce concentrations of chlorpyrifos below the University of California Davis (UC Davis) aquatic life water quality criterion of 10 ng/L [35] because the detection limit of the analytical method was 50 ng/L. The VTS in the current study reduced concentrations of permethrin below the UC Davis acute criterion of 10 ng/L in five of six simulated irrigation trials, and below the chronic criterion of 2 ng/L in one trial [36]. The VTS was not able to reduce imidacloprid concentrations below the UC Davis acute criterion of 170 ng/L [37]. The expanded VTS used in the second phase of the study, which included a sediment trap and the application of PAM, reduced permethrin concentrations below the chronic criterion in two of three irrigation events monitored, and was able to reduce the imidacloprid concentrations below the acute criterion in one irrigation event.

Although concentrations of imidacloprid greater than 4 µg/L were reduced to below 1 ng/L in laboratory experiments with biochar [21], the field installations in the current study were unable to thoroughly remove the insecticide. This treatment inefficiency was likely because of water bypassing the biochar installations. Further study is needed to improve the design of the biochar filter so that a majority of water passing through the system comes in contact with the carbon. Some of this work is already being conducted in the Salinas Valley, CA, through the construction of flumes that direct water through the mesh-encased biochar (Parry Klaussen, CARES, personal communication) or through the use of more loosely packed biochar mixed with other materials (Brant Knopp, Leland Environmental, personal communication). Preliminary data from a low-flow system that behaves more like the columns used in laboratory experiments show the most promise for complete pesticide removal (Marine Pollution Studies Laboratory, unpublished data).

To meet regulatory water quality targets and protect receiving systems, growers will need to implement strategies that reduce chemical and particle loads in runoff. There are a number of available practices ranging from source control to the integrated system discussed above. These systems can often provide near-complete removal of pesticides and associated toxicity if they have a complete suite of components, including retention basins and sediment traps, vegetated water ways, treatment with PAM, and activated carbon or biochar to sequester water soluble pesticides [1]. Designs that address the treatment of both water soluble and hydrophobic pesticides are necessary for complete treatment to minimize toxicity from agricultural discharges.

## Figures and Tables

**Table 1:** Inflow and outflow water volumes, insecticide and total suspended solid concentrations, and relative concentration (Conc.) and load reductions. ND indicates non-detect.

Carbon Type	Granulated Activated Carbon			Biochar		
**Date**	**25/3/2019**	**22/4/2019**	**14/5/2019**	**8/4/2019**	**15/4/2019**	**20/5/2019**
Inlet (Liters)	55,145	53,022	58,593	64,430	52,643	52,150
Outlet (Liters)	28,895	12,814	14,898	16,335	12,253	16,335
% Infiltration	48	76	75	75	77	69
**Imidacloprid**						
Inlet (ng/L)	3220	3210	4080	4790	3520	4090
Pre-Carbon (ng/L)	935	1770	1790	1510	1530	1840
Outlet (ng/L)	382	377	342	270	370	315
Reduction in Conc. (%)	−88	−88	−92	−94	−89	−92
Reduction in Load (%)	−94	−97	−98	−99	−98	−98
**Permethrin**						
Inlet (ng/L)	164	218	266	296	271	285
Pre-Carbon (ng/L)	2	29	11	39	35	36
Outlet (ng/L)	ND	19	5	4	3	5
Reduction in Conc. (%)	−100	−91	−98	−99	−99	−98
Reduction in Load (%)	−100	−98	−100	−100	−100	−99
**Total Suspended Solids**						
Inlet (mg/L)	107	194	158	295	189	139
Pre-Carbon (mg/L)	60	30	22	29	32	54
Outlet (mg/L)	75	61	28	47	29	81
Reduction in Conc. (%)	−30	−69	−82	−84	−85	−42
Reduction in Load (%)	−63	−92	−95	−96	−96	−82

**Table 2:** Toxicity test results and related imidacloprid and permethrin toxic unit (TU) values based on insecticide concentrations measured in simulated runoff. Shaded cells indicate significant toxicity or toxic unit values greater than 0.5.

	Granulated Activated Carbon			Biochar		
	25/3/2019	22/4/2019	14/5/2019	8/4/2019	15/4/2019	20/5/2019
***H. azteca*** **Survival**						
Inlet (%)	0	0	0	2	0	0
Pre-Carbon (%)	0	2	48	2	0	0
Outlet (%)	0	8	96	48	0	0
***H. azteca*** **Toxic Units**						
Imidacloprid						
Inlet	0.05	0.05	0.06	0.07	0.05	0.06
Pre-Carbon	0.01	0.03	0.03	0.02	0.02	0.03
Outlet	0.01	0.01	0.01	0.00	0.01	0.00
Permethrin						
Inlet	7.8	10.3	12.6	14.0	12.8	13.5
Pre-Carbon	0.12	1.36	0.51	1.84	1.65	1.69
Outlet	0	0.89	0.22	0.17	0.15	0.25
***C. dilutus* Survival**						
Inlet (%)	40	38	0	6	4	0
Pre-Carbon (%)	85	96	90	77	96	0
Outlet (%)	94	98	99	83	93	0
***C. dilutus* Toxic Units**						
Imidacloprid						
Inlet	0.56	0.56	0.71	0.83	0.61	0.71
Pre-Carbon	0.16	0.31	0.31	0.26	0.27	0.32
Outlet	0.07	0.07	0.06	0.05	0.06	0.05
Permethrin						
Inlet	1.66	2.20	2.69	2.99	2.74	2.88
Pre-Carbon	0.02	0.29	0.11	0.39	0.35	0.36
Outlet	0	0.19	0.05	0.04	0.03	0.05
***C. dilutus* Growth**						
Inlet (mg)	0.03	0.02	NA	0.1	0.10	NA
Pre-Carbon (mg)	0.81	4.07	2.19	2.74	1.71	NA
Outlet (mg)	5.35	5.43	3.40	2.93	5.79	NA

**Table 3:** Inflow and outflow water volumes for the 12 November trial, as well as insecticide and total suspended solid concentrations, and relative concentration and load reductions for all trials. Load reductions for the 24 September and 8 October trials were estimated (*) based on flow measurements taken during the 12 November trial. ND indicates non-detect. NR indicates not recorded due to equipment malfunction.

**Date**	**24/9/2019**	**8/10/2019**	**12/11/2019**
Inlet (L)	NR	NR	58,211
Outlet (L)	NR	NR	3309
% Infiltration	NR	NR	94
**Imidacloprid**			
Pre-Trap (ng/L)	912	546	22,800
Inlet (ng/L)	1090	422	23,600
Pre-Carbon (ng/L)	317	314	7580
Outlet (ng/L)	186	135	5860
Reduction in Conc. (%)	−80	−75	−74
Reduction in Load (%)	−99 *	−98 *	−99
**Permethrin**			
Pre-Trap (ng/L)	42.6	32.8	73.4
Inlet (ng/L)	26.6	16.5	30.3
Pre-Carbon (ng/L)	ND	ND	10.7
Outlet (ng/L)	ND	ND	15.9
Reduction in Conc. (%)	−100	−100	−48
Reduction in Load (%)	−100 *	−100 *	−97
**Total Suspended Solids**			
Pre-Trap (mg/L)	424	249	282
Inlet (mg/L)	265	100	179
Pre-Carbon (mg/L)	101	45	66
Outlet (mg/L)	77.0	41	61
Reduction in Conc. (%)	−82	−84	−78
Reduction in Load (%)	−98 *	−98 *	−98

**Table 4:** Results of toxicity tests and related imidacloprid and permethrin toxic unit values based on insecticide concentrations measured during lettuce field runoff events. Shaded cells indicate significant toxicity or toxic unit values greater than 0.5.

Date	24/9/2019	8/10/2019	12/11/2019
***H. azteca* Survival**			
Pre-Trap (%)	0	22	0
Inlet (%)	0	40	0
Pre-Carbon (%)	24	90	29
Outlet (%)	76	96	56
***H. azteca* Toxic Units**			
Imidacloprid			
Pre-Trap	0.01	0.01	0.35
Inlet	0.02	0.01	0.36
Pre-Carbon	0.00	0.00	0.12
Outlet	0.00	0.00	0.09
Permethrin			
Pre-Trap	2.02	1.55	3.48
Inlet	1.26	0.78	1.44
Pre-Carbon	0	0	0.51
Outlet	0	0	0.75
***C. dilutus* Survival**			
Pre-Trap (%)	0	21	0
Inlet (%)	0	90	0
Pre-Carbon (%)	0	96	0
Outlet (%)	75	92	0.02
***C. dilutus* Toxic Units**			
Imidacloprid			
Pre-Trap	0.16	0.09	3.97
Inlet	0.19	0.07	4.10
Pre-Carbon	0.06	0.05	1.32
Outlet	0.03	0.02	1.02
Permethrin			
Pre-Trap	0.43	0.33	0.74
Inlet	0.27	0.17	0.31
Pre-Carbon	0	0	0.11
Outlet	0	0	0.16
***C. dilutus* Growth**			
Pre-Trap (mg)	NA	0.10	NA
Inlet (mg)	NA	1.89	NA
Pre-Carbon (mg)	NA	2.18	NA
Outlet (mg)	0.72	5.03	0.2

## Data Availability

The data presented in this study are available on request from the corresponding author. The agencies involved in this study have no mechanism to make the data publicly available.
